# Information management to enable personalized medicine: stakeholder roles in building clinical decision support

**DOI:** 10.1186/1472-6947-9-44

**Published:** 2009-10-08

**Authors:** Gregory J Downing, Scott N Boyle, Kristin M Brinner, Jerome A Osheroff

**Affiliations:** 1 Personalized Health Care Initiative, United States Department of Health and Human Services, Washington, DC, USA; 2 Thomson Reuters, Greenwood Village, CO, USA; 3 University of Pennsylvania Health System, Philadelphia, PA, USA

## Abstract

**Background:**

Advances in technology and the scientific understanding of disease processes are presenting new opportunities to improve health through individualized approaches to patient management referred to as personalized medicine. Future health care strategies that deploy genomic technologies and molecular therapies will bring opportunities to prevent, predict, and pre-empt disease processes but will be dependent on knowledge management capabilities for health care providers that are not currently available. A key cornerstone to the potential application of this knowledge will be effective use of electronic health records. In particular, appropriate clinical use of genomic test results and molecularly-targeted therapies present important challenges in patient management that can be effectively addressed using electronic clinical decision support technologies.

**Discussion:**

Approaches to shaping future health information needs for personalized medicine were undertaken by a work group of the American Health Information Community. A needs assessment for clinical decision support in electronic health record systems to support personalized medical practices was conducted to guide health future development activities. Further, a suggested action plan was developed for government, researchers and research institutions, developers of electronic information tools (including clinical guidelines, and quality measures), and standards development organizations to meet the needs for personalized approaches to medical practice. In this article, we focus these activities on stakeholder organizations as an operational framework to help identify and coordinate needs and opportunities for clinical decision support tools to enable personalized medicine.

**Summary:**

This perspective addresses conceptual approaches that can be undertaken to develop and apply clinical decision support in electronic health record systems to achieve personalized medical care. In addition, to represent meaningful benefits to personalized decision-making, a comparison of current and future applications of clinical decision support to enable individualized medical treatment plans is presented. If clinical decision support tools are to impact outcomes in a clear and positive manner, their development and deployment must therefore consider the needs of the providers, including specific practice needs, information workflow, and practice environment.

## Background

In the coming years, electronic health records (EHR) will have increasingly important roles in the delivery of health care, particularly in creating new opportunities to improve quality and effectiveness. Among the most important features offered by EHR are electronic exchange and interoperability of patient health information; new tools for carrying out some care delivery functions, like drug prescribing; supporting providers in the delivery of evidence-based care, including incorporation of evolving practice guidelines into point-of-care accessible information formats; measurement of quality in care delivery; and widespread access to large networks of data for research and quality purposes. While improvements in quality of care involve all of these functions, it is particularly in the area of clinical decision support (CDS) that EHR-based technology is expected to improve delivery of care in a continually evolving manner.

There will be an interdependent relationship between CDS and personalized medicine in the effort to achieve improvements in the quality of health care. CDS will help health care providers cope with an increase in the number of clinical decisions and acceleration of knowledge development that would not be manageable without EHR systems. As medical knowledge increasingly steers toward variability in treatments based on individual patient factors, including genomic-related clinical variables, the need for CDS as a central part of care delivery will grow. Thus, improving quality of care is expected to involve increasing numbers of options for therapy, based on increasingly detailed amounts of quantitative data and knowledge, and resulting in increasing precision and effectiveness of care for each patient. For the most part, CDS tools today do not provide support for computing of complex quantitative determinations such as risk assessment, determination of combinatorial therapeutic interventions, and prediction of outcomes from interventions. Improvements in CDS development will be essential to achieving measurable advances in clinical outcomes in scenarios where health care providers are faced with increasing numbers of clinical variables.

Today, even as the basic foundation of EHR systems is being laid, the pathways for CDS development are simultaneously being designed. This task is complex in itself, and it is made even more complex by the need for CDS design to anticipate and accommodate the expected growth of differentiation in patient care - i.e., personalized medicine. In this paper, we examine the potential and current state of CDS development; we identify roles for key stakeholders involved in the various elements of CDS design and construction; and, we recommend steps the various stakeholders can take at this stage to enable the long term goal of CDS support for personalized medicine.

## Discussion

### Future Applications of Clinical Decision Support in Personalized Medicine

While there are many contexts for which the term 'personalized medicine' can be applied, we take a broad view of its future applications. Personalized medicine is defined as the delivery of health care in a manner that is informed by each person's unique clinical information; genetic, genomic, and other molecular/biological characteristics; and environmental influences. The goals of personalize medicine are to take advantage of a molecular understanding of disease, combined with other individual factors, to optimize preventive health care strategies while people are still well or at the earliest stages of disease. [1]

In 2007, the American Health Information Community (AHIC) established a work group to address standards and interoperability specifications to enable EHR systems to exchange and apply genomic information in medical decision-making. CDS was among the four priority areas targeted for workgroup activity in large part because of the complexity that individualized patient care delivery presents to health care providers' practices. [2] Through detailed analysis of existing resources and testimony provided in public meetings, the workgroup found that there is no systematic process to develop, disseminate, and incorporate evidence-based practice information within the clinical community. As a consequence, the findings indicated that it may take years to develop and incorporate guidelines into daily clinical practice through electronic systems. The workgroup indicated the importance of clarifying the needs and opportunities for integrating CDS tools to address personalized medicine as EHR systems begin to offer decision support capabilities and adoption of their use increases. Further, the workgroup found it useful to align the needs with existing capabilities by organizations or stakeholders in CDS development to support personalized medicine practices.

The completion of the Human Genome Project, followed by the development of a more manageable understanding of the human genome in the Hap Map project and the launch of genome-wide association studies (GWAS), marked a great accomplishment and initiated a burst in scientific discovery of the genetic underpinnings of common diseases. [3] This is the basis for many current efforts to enhance preventive care strategies, improve diagnosis, avoid adverse events, and inform appropriate selection and dosing of drug therapies using diagnostic and predictive molecular tests. The last two decades have also brought a shift toward a large number of molecularly-targeted therapies, advanced imaging tools, and cell- and immune-based therapies which are designed for use to meet specific characteristics of each patient's disease course. Insights gained from this research now show that genomic information is leading to meaningful sub-stratification of a wide array of disease conditions that enables prediction an individual's of risk for disease and the likelihood of an effective or unsafe response to therapy.

Realization of personalized medicine is dependent on the ability to collect, disseminate, and process complex information in the context of clinical care. The traditional paradigm of a provider reading current literature and submitting adverse event reports cannot support this complexity. It requires an EHR infrastructure to provide access to key clinical data with CDS capabilities that provide the right information, at the right time, at the point-of-care.

CDS tools have the potential to enable personalized approaches to health care by providing health care providers, their staff, and patients with information and preferences specific to the individual, intelligently filtered and combined at appropriate times, to enhance health and health care. Primary methodologies for CDS include information retrieval; evaluation of logical conditions; probabilistic and data-driven classification or prediction; heuristic modeling systems; calculations, algorithms, and multi-step processes; and associative groupings of elements. [4] CDS encompasses, but is not limited to, computerized alerts and reminders to providers; methods to bring care into compliance with clinical guidelines; generation of order sets, patient data reports and summaries, and documentation templates; advice to promote more accurate and timely diagnoses; and tools that enhance clinical workflow. [5] Although deployed inconsistently in health care at present, CDS tools may support a variety of processes to improve health care quality by engaging the patient in the decision-making process. [6] These resources may also increase providers' effectiveness by enhancing their ability to use a greater array of information to improve the quality of care, avoid adverse events, provide actionable guidelines, and efficiently integrate newly-discovered information into clinical practice, thereby enabling informed patient-provider interactions and facilitating predictable outcomes in health care strategies. [7-9] CDS tools also enable application of performance measures to practice: that is, tools that can embed evidence-based treatment guidelines can also help to assess practice-based quality improvement programs and measure clinical outcomes. For CDS tools being used to integrate multiple clinical variables and genetic test results, clinical supporting documentation for the decision options should be transparent to the user to ensure that the scientific basis for the recommended actions are suitable for the patient of interest.

In summary, CDS tools will likely become critical components of personalized medicine paradigms, which rely on EHR capabilities to assess a variety of data types, including an individual's genetic/genomic data, within evidence-based guidelines and practices. As the individualized information is accumulated and the applications of tailored treatment decisions expand, it is likely that providers and patients will need CDS tools to reap the benefits from the information influx. When combined with the provider's clinical judgment, these tools will complement non-EHR-based data sources (e.g., reference datasets from genomic databases, laboratory reference sets, and web-portals) to offer an additional resource to assist with clinical decision-making.

The following commonly occurring clinical scenarios compare and contrast current and future applications of information support to facilitate individualized decision-making. In addition, these examples demonstrate the gaps in capabilities and can serve as a springboard for recommendations to close them. These scenarios represent the current knowledge base and are reflective of the types of genetically-based information used in decision-making today.

#### Example 1. Application of viral genotyping in therapeutic decision-making

##### Current state of antiretroviral treatment for human immunodeficiency virus (HIV) infection

Patient X commences antiretroviral treatment after testing positive for HIV. His health care provider prescribes a non-nucleoside reverse transcriptase inhibitor (NNRTI) and concurrently orders a genotype resistance test to identify HIV-reverse-transcriptase-based mutations, as certain variants may decrease the effectiveness of the prescribed inhibitor. However, despite the identification of the K103N mutation in the reverse transcriptase, which is broadly accepted to confer definitive resistance, [10] the treatment course is not altered. As a result, the patient does not achieve full antiviral suppressive effect.

While it may seem difficult to imagine that a health care provider would order a resistance panel and then seemingly ignore its results, the scenario is more complex than this simple analysis. As with many rapidly-advancing fields, the consensus on genotype is constantly evolving, as is the health care providers' understanding of and currency with this knowledge. In a recent study concerning HIV genotypic resistance testing and antiretroviral prescription practices in a group of experienced HIV health care providers, researchers analyzed the frequency with which patients were prescribed antiretroviral therapies that were inconsistent with viral genotypic information. [11] Among patients in this group, 18% continued to be treated with an antiretroviral definitively demonstrated to be resistant to the prescribed medication. In nearly one-third of these instances, prescribers reported that these actions were due to "erroneous oversights," suggesting that CDS capabilities such as electronic prescription (e-prescription) software integrated with genotypic test ordering (see below) and appropriate information for interpretation of results could reduce the incidence of such inappropriate action.

##### Future state of antiretroviral treatment for HIV

Patient Y commences antiretroviral treatment after testing positive for HIV. Using e-prescription software embedded within the EHR system, his health care provider prescribes an NNRTI and is concurrently prompted to order a genotype resistance test to identify mutations that may decrease the NNRTI's effectiveness; the order is added with a single click from the alert screen. The results are returned, and the test identifies the K103N mutation in the reverse transcriptase. The automated electronic patient record system cross-references the test result with the current prescription, notifies the provider about the patient's resistance to the current medication, and suggests several alternate NNRTIs that are not contraindicated by this genotypic result. The tool provides the physician with an "information button" in the EHR that provides information that augments the decision to change medication. The physician makes thus makes medication changes (with the help of tools similar to the one that facilitated the test ordering), and the patient achieves the desirable reduction in viral load.

#### Example 2. Augmenting health risk determination using CDS tools

##### Current state of estimating invasive breast cancer risk

Patient X has a family history of breast cancer and initiates a conversation with her provider to discuss risk prediction and preventive strategies. The provider utilizes a web-based tool or user-installed program on her personal digital assistant device such as the National Cancer Institute's Breast Cancer Risk Assessment Tool [12] (based on the Gail Model Breast Cancer Reduction Tool) that estimates the patient's risk of developing invasive breast cancer during the next 5-year period and up to age 90 years (lifetime risk). This risk assessment is based on family, medical, and reproductive history that must be manually entered into the tool. The patient and the provider discuss the results generated by the tool, and each receives a printed copy of the results.

##### Future state of estimating invasive breast cancer risk

An EHR contains standardized family health history, medical history, laboratory results (including genomic information from previous testing), and other clinical information. In addition, the EHR has embedded risk-prediction tools, which can utilize more complex information sources and do not require re-entry of data that are contained within the patient's medical record. As the patient enters various life stages for which risk factors are modified or updated, the risk determination is actively revised and provided to the patient and provider, along with personalized information and guidance on interpreting and addressing the information provided. For example, on her 35^th ^birthday, Patient Y receives notification that she should schedule an appointment with her provider to discuss risk prediction and other preventive strategies for breast cancer. This notification is generated based on recommendations from evidence-based practice guidelines. However, when combining this with patient-specific information in the EHR, the provider can receive an automatically-generated message that details the patient's updated risk assessment and spur a discussion with the patient during a routine health visit. Because of the automated information delivery and the detailed information provided in an electronic CDS environment, patients gain knowledge about health risks while providers are offered information (and potentially a greater range of complementary, pertinent information) more efficiently.

As demonstrated above, CDS tools will enable health care providers and patients to more efficiently and effectively gather, interpret, process, and apply an increasing volume of complex information needed for up-to-date, best-practice care. However, for CDS to be effective in managing decisions often encountered in primary care, such as diabetes, heart disease, or mental illness, algorithms must integrate an increasing array of variables to tailor an accurate and useful risk probability assessment to a given patient. In addition, mitigating factors such as co-morbidities and non-mutation-based genomic changes occur over time and may create additional considerations when developing an intervention. When one considers that multiple gene combinations may suggest different management paradigms for a given condition, the landscape becomes quite complex.

### Challenges to the Adoption and Use of CDS Tools

The impact of EHRs and related platforms (such as personal health records) on patient outcomes has been inconsistent and difficult to quantify. [7] While individual institutions have demonstrated that locally-developed HIT systems can improve health care quality and efficiency [13] and can improve care for some chronic illnesses, [14] incorporating interoperable CDS resources into widely-disseminated health IT platforms remains challenging for several reasons. First, these resources must recognize evolving technologies, such as genomic assays, that have yet to matriculate fully in clinical care. As the *BRCA *(genes associated with hereditary breast cancer) scenario reveals, the use of genetic and genomic tests, when coupled with predictive risk assessments provided by family health history information, offers tremendous potential to deliver timely, appropriate prevention and care. However, it also adds complexity to the decision-making process that will only increase as additional genomic information becomes validated.

In addition, interoperable tools must be founded on an agreed-upon baseline set of evidence-based best practices that exclude ad-hoc, vendor-specific, or experiential rules. Third, formal medical education typically provides minimal training in applying genetic/genomic tests and data, limiting many health care providers' abilities to interpret this information. Furthermore, constraints and demands of current clinical practice often discourage the convenient acquisition of this knowledge. To appropriately interpret results, the provider must seek information from external sources, such as the laboratory performing the tests, knowledge repositories, or genetic specialists. Currently, this process is inefficient, and the burden for improved information-handling will increase, particularly among primary care practices, as more genomic information is integrated into the health care system. If CDS tools are to impact outcomes in a clear and positive manner, their development and deployment must therefore consider the needs of the provider, including specific practice needs, information workflow, and practice environment.

Compared to paper-based systems, interoperable CDS resources offer the potential to transfer information more rapidly and perhaps more reliably, however much work remains to be done before these resources become standard practice. Wide deployment of CDS tools supporting clinical medical decision making will be facilitated through standardization (agreed-upon standards for the rules and data fields that will enable the various electronic resources to utilize various data types and resources) and interoperability (the capability of an information system to exchange data with other systems). Data from different sources, including EHRs, claims databases, genomic databases, and laboratory results files, are used in various ways. Standards and interoperability enable information in each of these resources to be related to each other, creating new knowledge from their integration. Currently, this goal is hampered by the challenges of incorporating proprietary technologies that have developed over time in existing EHR systems with emerging applications and capabilities. Even widely-used predictive algorithms, such as the National Cancer Institute's Breast Cancer Risk Assessment Tool [12] and the National Heart, Lung, and Blood Institute's "Framingham" risk assessment tool for coronary heart disease, [15] are not always associated with EHRs. Likewise, acceptance of automatically-deployed alerts in clinical practice is constrained by lack of information regarding which interventions (such as diagnostic tests, devices, and therapies) work in given situations, how users react to CDS-deployed messages, and how these tools affect outcomes. As a result, there is a growing need to understand how to incorporate these tools into clinical workflows to meet the needs of providers and patients.

As CDS becomes incorporated into EHRs, additional efforts are required, both in research and application, before these tools can be optimized for personalized disease management at the point-of-care. Appropriate oversight can facilitate the integration process by promoting interoperable standards, assisting in the development of quality control metrics for information used in CDS tools, and assessing the impact of the tools on outcomes and performance measures. The recommendations discussed in this paper offer a dialogue to create avenues that will ultimately facilitate the adoption and use of CDS tools in medicine.

### Stakeholders' Roles in Supporting Individualized Approaches to Health Care

The pathway to use CDS in support of personalized medicine requires many integrated components to be developed in parallel with the emerging EHR foundation in health care. Developing a health care system that optimizes personalized prevention and treatment requires engagement from many diverse stakeholders. Organizing the work that needs to be done by stakeholder organizations is a helpful framework to consider the actors and actions needed for the complete picture of personalized medicine CDS systems to emerge. These include the consumer/patient, molecular diagnostic (laboratory test or device) developer, providers, payor, CDS developers, and oversight and regulatory bodies, among many others.

**Figure 1:**
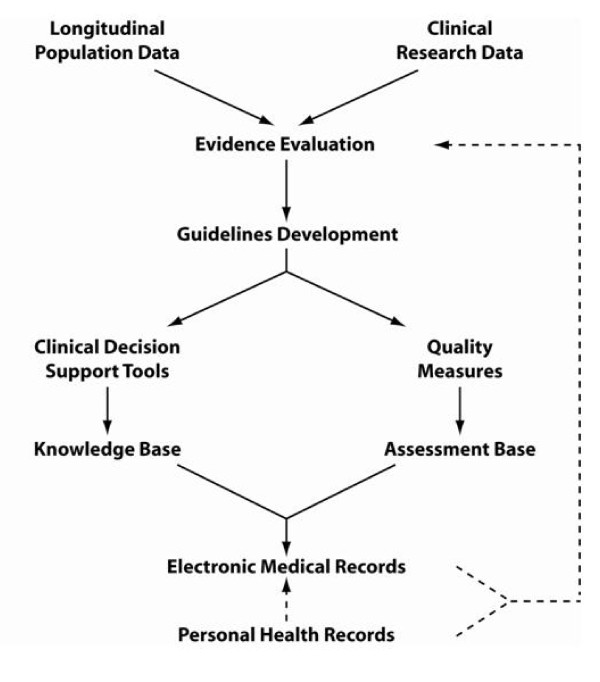
**The role of CDS tools and electronic and personal health records in the development of clinical practice guidelines and quality measures**. (Solid lines indicate currently available information flow patterns, dotted line represents potential pathways for future data flow).

An overview of the continuum of information flow from evidence development through clinical application using EHR technology is shown in Figure 1. Personalized medical practices will increasingly be based on scientific evidence gained from population-based longitudinal studies and clinical research studies such as randomized clinical trials. These inputs provide the evidence that certain medical technologies are recommended or shown to have benefit under various clinical conditions. This becomes the basis for algorithms or treatment recommendations that are integrated into practice guidelines for many medical conditions to be used in patient care encounters in two major applications. First, key program inputs are created for CDS tools and integrated into electronic knowledge base that is used to support the rules used in making recommendations to providers for individualized treatment decisions. Second, the guidelines are increasingly used in formulating quality measures to improve compliance in medical decision-making and further assessment of provider and institutional compliance with recommended medical practices. EHR systems will provide the framework for supporting the appropriate use of guidelines in medical decision-making and a means for evaluating performance based health outcomes relative to clinical care quality measures. Ultimately, health care quality improvement measures will integrate over time information from EHRs and potentially personal health records in a feedback mechanism for guideline improvements. This framework presents an operational view of the key steps in information development needed for CDS to support personalized medical practice.

To address the information needs outlined in the framework above, this section discusses the key steps and which stakeholders will likely be best suited to address them. A summary of recommendations for the various stakeholders is provided in Table 1. Much of the required work to achieve CDS to support individualized medical practice is not fundamentally different from other applications. However, there are key aspects needed to support individualized approaches.

**Table 1:** Supporting Individualized Health Care: Recommendations for Stakeholders

*Stakeholder*	*Recommendations*
Government	* Support development of a minimum data set of personal attributes that contribute to individualized care * Set national priorities for health care quality improvement driven by the National Quality Forum * Identify priorities for Federally-funded CDS efforts * Evaluate the impact of these programs on high-priority areas by representatives of Federal agencies * Work with HITSP [31] to identify standard data types needed to support CDS tools of high-priority conditions determined by national priority-setting efforts * Coordinate with CCHIT [32] on certification criteria for CDS to enable support of personalized medical practices * Expand research support to promote best practices for utilizing CDS tools in EHR systems

Researchers and Research Institutions	* Engage professional societies and EHR vendors to understand the types of information necessary to integrate new CDS tools into health care * Explore methodologies to present tools to providers * Assess patient-provider interfaces * Evaluate the role and impact of electronic tools in shared decision-making * Support innovative software applications that integrate multiple variables (e.g., test results, medical/medication history, patient preferences, family health history).

Developers of Electronic Information Tools	* Create tools to identify appropriate molecular diagnostic tests, provide additional background material or references, or produce a personalized interpretation based on existing medical data at the point-of-care * Build tools to support workflow integration for diagnostic test orders, integration of test results into confirmatory tests, intervention options, and patient information resources.

Developers of Clinical Practice Guidelines (CPG) and Quality Measures (QM)	* Include patient-specific factors for disease management when supported by an evidence base (CPG) * Incorporate differences among subsets of patients (CPG) * Translate practice guidelines into machine-readable formats to integrate into CDS tools (CPG) * Work with electronic tool developers to create standard data formats that can be used to structure guidelines for import and use in CDS formats (CPG) * Work with professional societies to design measures that incorporate into EHRs (QM) * Harmonize measure specifications with EHR standards and requirements (QM) * Ensure that EHRs are equipped to capture dataset to evaluate quality measures (QM).

Developers of EHR Standards	* Encourage standards development for integrating practice guidelines into CDS tools and with EHR systems * Prioritize genetic test and family history application standards development and certification processes * Foster development of evaluation criteria for the usability and effectiveness of CDS tools in EHR systems.

### Multi-Sector Collaborations

Integrating CDS tools into clinical practice will require multi-layered policy interventions to overcome systemic barriers and challenges currently facing personalized medicine. [16,17] In 2005-06, the HHS Office of the National Coordinator for Health Information Technology (ONC), [18] in partnership with the Agency for Healthcare Research and Quality (AHRQ), supported the development of a National CDS Roadmap. [5] Coordinated by the American Medical Informatics Association, the Roadmap activity convened experts in informatics, software engineering, and evidence development from industry, academia, and Federal agencies to develop a national plan of action for CDS. The Roadmap identified three essential elements for achieving the promise of CDS in health care: 1) access to the best knowledge available; 2) widespread adoption and effective use of CDS tools; and 3) continuous improvement of knowledge and CDS methods. This plan represents a multi-sector strategy to advance the development and application of decision-support capabilities to enable individualized patient management, thereby bridging the gap between current and future approaches to CDS.

### Government

Numerous government agencies currently participate in initiatives that support information needs for personalized medical practices including the Department of Health and Human Services' (HHS) Personalized Health Care Initiative, [19,20] the AHRQ-sponsored information resources developed by the U.S. Preventive Services Task Force (USPSTF), [21] and the Centers for Disease Control and Prevention's Evaluation of Genomic Applications in Practice and Prevention (EGAPP) initiative. [22] Through 2008, federal health information technology activities were coordinated through the American Health Information Community (AHIC), [23] which was established in 2005 to harmonize standards and to improve interoperability and networking applications that will accelerate the adoption of EHR systems and, ultimately, enable the development of a nationwide interoperable EHR infrastructure. ONC provides support for the AHIC and coordinates HHS activities. During 2007, several AHIC Workgroups identified CDS capabilities as a timely and important area of focus, and a CDS Ad Hoc Planning Group developed formal recommendations (Table 1) to accelerate the implementation of robust and workflow-sensitive CDS interventions that will drive measurable improvement in key health care outcomes. [24] A multi-stakeholder CDS Collaboratory, co-sponsored by AHRQ, the HHS Personalized Health Care Initiative, and ONC, has been formed to coordinate internal federal efforts. This group will build upon an assessment of CDS-related federal agency activities conducted in 2007 to leverage collective efforts and knowledge to expedite development and adoption of effective CDS capabilities.

Among the priorities to enhance CDS development for future personalized medicine approaches is the establishment of EHR standards for a minimum data set of key variables. This minimum data set would include demographic data, physical measures, family health history, medications, health risk factors, and other parameters. CDS tools can then be built through rules and algorithms deployed through EHR systems using data supported from the patient's EHR. Other priorities include establishing pathways for integrating requirements for quality measures and standards and certification with the primary organizations responsible for them.

### Researchers and Research Institutions

CDS resources will accelerate the integration of new knowledge and technologies into health care practice. Standards development can orient this information towards appropriate end-users, but only if the needs and the output of those users are well understood. As such, the development of optimal CDS tools will require input from basic, clinical, and outcomes researchers. As new information that may personalize medical care emerges, researchers should consider engaging with professional societies and EHR vendors to understand the types of information that will be necessary to integrate the new tools into care. Many current clinical systems include mechanisms to provide providers with relevant, trusted information as they make clinical decisions. As new tools are integrated, however, researchers should explore methodologies to present tools to providers, assess patient-provider interfaces, evaluate the impact of electronic tools in shared decision-making, and support innovative software applications that integrate multiple variables such as test results, medical history, medication history, patient preferences, and family health history. Current research on the use of CDS tools in quality improvement measures, workflow, and shared decision-making practices should continue. [25] For example, a new initiative sponsored by AHRQ focuses on the development, adoption, implementation, and evaluation of best practices using CDS tools integrated within EHR, [26] and integrated health care delivery systems, such as the Geisinger Health System, are developing and applying CDS tools that include patient preference modules within their electronic health environments. [27]

### Developers of Electronic Information Tools

Developers of electronic information tools can advance personalized medicine by creating tools to identify appropriate molecular diagnostic tests, provide additional background material or references, or produce a personalized interpretation based on existing medical data at the point-of-care. When setting priorities, vendors should build tools to support medical decision-making, including workflow integration for diagnostic test orders, integration of test results into confirmatory tests, intervention options, and patient information resources. High-impact areas needed for development include tools for detecting potential adverse events, genetic assay support for treatment selection, and reminder systems for risk detection, disease screening, and prevention.

These tools may capitalize on established EHR technologies such as e-prescribing. For example, by augmenting the information provided to pharmacies, the pharmacist could become a resource providing assurances for patient safety, minimizing adverse events, and improving health outcomes. Recent initiatives advanced by the Centers for Medicare and Medicaid Services are providing financial incentives for e-prescribing, and recent legislative action is providing a stimulus incentive to physicians to use this technology. [28] Providing pharmacists with clinical data attributes (e.g., allergy status, pharmacokinetic data, pharmacogenomic results or interpretations) can facilitate communication, verify proper dosing decisions, and enhance consumer education. In addition, including CDS in e-prescribing systems may improve the safety, quality, efficiency, and cost-effectiveness of care [29] and permit providers to leverage standard-of-care guidelines. Developing tools to augment personalized medical decisions will help avoid adverse events and improve decision-making on dose and therapy selection that can be informed by genetic or other molecular diagnostic testing. As technology improves and greater strides are taken toward use of CDS to improve performance against patient-care quality measures, similar incentives may be considered to accelerate the use of computer-assisted decision-making. New techniques and educational efforts will likely be needed to help care providers to integrate use of CDS tools into workflow and patient-management paradigms.

### Organizations that Develop Clinical Practice Guidelines and Quality Measures

Evidence-based practice guidelines offer the health care provider a general reference framework from which to choose interventions to address a patient's health care needs. However, no two patients are alike, and an individual's unique genomic/biological profile and personal preferences may suggest appropriate treatment avenues that differ from those applicable to other individuals who manifest the same disease. Practice guidelines should include patient-specific factors for disease management when these considerations are supported by an evidence base. Given the multi-factorial nature of chronic conditions commonly encountered in primary care (e.g., type 2 diabetes, cardiovascular diseases, dyslipidemias, depression), however, it is reasonable to expect increasingly nuanced treatment algorithms to emerge. To benefit patients fully, clinical practice guidelines and quality measures must incorporate differences among subsets of patients as the relationships between molecular profiles and treatment response are elucidated. As mentioned previously, CDS tools have the potential to identify diagnostic tests and other criteria to stratify patients and identify the appropriate clinical guidelines for a given subset of patients. In turn, these tools can inform quality improvement efforts at the level of the practice and quality metrics related to policy development and education. However, practice guidelines must be translated into machine-readable formats to integrate into CDS tools, and it is recommended that organizations that develop practice guidelines work with electronic tool developers to create standard data formats that can be used to structure guidelines so that they can be imported and used in CDS formats.

Recent legislation, such as the Medicare Improvements for Patients and Providers Act of 2008 [28] and American Recovery and Reinvestment Act of 2009 [30], identifies the need for a consensus-based processes to prioritize, endorse, and maintain performance measures and use them with EHR systems to provide meaningful improvements in health care practices. As national priorities for health care quality are established by quality alliances and other health care stakeholders (e.g., consumers, purchasers/employers, providers, accreditation programs, insurers, governmental and other organizations), the effects of individualized approaches on treatment decisions and procedures must be considered. Based on potential challenges to incorporate individualized approaches to care into clinical guidelines and quality measures, specialty societies, health systems, and other groups with longitudinal databases that develop clinical guidelines should harmonize their guidelines and engage CDS tool developers. These harmonized guidelines should leverage evidence for stratifying patients with all types of biological differences and incorporate individualized approaches to clinical care. In addition, EHRs and CDS tools may provide a near real-time data stream for guideline developers and clinical researchers to collect performance measurement data and refine the evidence base, practice guidelines, and procedural and quality measures. For example, standardized exclusion lists within the EHR may provide data to explain deviations from a guideline, such as contraindication because of genetic or other interaction or patient may have opted out of treatment. Thus, developers of quality measures are strongly encouraged to work with the vendor community to design measures that incorporate into EHRs, harmonize measure specifications with EHR standards and requirements, and ensure that EHRs are equipped to capture dataset to evaluate quality measures.

### Organizations that Develop Health Information Technology Standards

The broad integration of complex, personalized information into the health care delivery process through CDS tools will require the development of standards to permit disparate entities (e.g., provider, clinical laboratory, patient, payer, pharmacy, research community) to utilize and exchange data. Considerations may include standardized terminology, metrics, and guidelines; examination of the workflow between various entities; identification of special datasets to collect (such as for safety monitoring or quality measures); and identification of policy and technical issues. EHR standards development organizations are encouraged to consider standards for templates that enable the integration of best practices into EHR systems. In addition, standards developers can recognize the value and potential of coupling genetic and family history information by prioritizing standards development through the Health Information Technology Standards Panel [31] and the Certification Council for Health Information Technology (CCHIT). [32] Recently, CCHIT announced plans to include CDS as an area of advanced technology that will be undergoing accelerated steps toward EHR certification standards in 2009-10. Standards-development organizations can also work to encourage evaluation measures for usability testing and performance of CDS tools in electronic information environments. These steps will help define value for users and create incentives for new tool development.

## Summary

Science, technology, and medical practice continue to enable innovative individualized approaches to health care that require sophisticated reasoning and decision making. The rate of development of these approaches will only accelerate, dramatically expanding patients' and providers' needs for tools to help process this information and take appropriate action. At present, electronic tools to support this approach to health care are used infrequently in EHR systems, yet these tools will be essential to support future point-of-care decision-making. As CDS development activities begin to unfold, assurances that these technologies will accommodate individualized approaches in medical decision-making will be essential for achieving more effective care through personalized medicine. Electronic information tool developers, researchers and research institutions, and standards development organizations will each have a role in bringing individualized approaches to health care, thereby effecting a transition that will benefit all health care stakeholders. It will require the efforts of numerous individuals and teams to develop the health care system that leverages individual differences for the prevention, early detection, and tailored treatment of human disease. Identifying key roles and activities across the health care enterprise can help accelerate the achievement of this goal for the benefit of patients and providers.

## Abbreviations

CDS: clinical decision support, EHR: electronic health records, HITSP: health information technology standards panel, CCHIT: certification commission for healthcare information technology, AHIC: American Health Information Community, GWAS: genome-wide association studies.

## Competing interests

JAO is chief clinical informatics officer of Thomson Reuters.

## Authors' contributions

Conceptual framework, organization, and content of the paper were developed equally by GD and JO. All authors contributed equally to the drafting, editing, and integration of comments from advisors. All authors read and approved the final manuscript.

## Pre-publication history

The pre-publication history for this paper can be accessed here:

http://www.biomedcentral.com/1472-6947/9/44/prepub

## References

[B1] Willard HW Willard HW, Ginsburg GS Organization, variation and expression of the human genome as a foundation of genomic and personalized medicine Genomic and Personalized Medicine 2009 1 London: Academic Press 4 21

[B2] Glaser J Henley DE Downing G Brinner KM Advancing Personalized Health Care through Health Information Technologyw: An Update from the American Health Information Community's Personalized Health Care Workgroup J Am Med Inform Assoc 2008 15 391 396 10.1197/jamia.M2718 18436899 PMC2442266

[B3] Manolio TA Brooks LD Collins FS A HapMap harvest of insights into the genetics of common disease J Clin Invest 2008 118 1590 1605 10.1172/JCI34772 18451988 PMC2336881

[B4] Greenes RA Greenes RA A brief history of clinical decision support: technical, social, cultural, economic, and governmental perspectives Clinical Decision Support: the Road Ahead 2007 Amsterdam, Elsevier 31 91

[B5] Osheroff J Teich J Middleton B Steen E Wright A Detmer D A roadmap for national action on clinical decision support J Am Med Inform Assoc 2007 14 141 145 10.1197/jamia.M2334 17213487 PMC2213467

[B6] Chaudhry B Computerized clinical decision support: will it transform healthcare? J Gen Intern Med 2007 23 suppl 1 85 87 10.1007/s11606-007-0432-9 PMC2150635 18095051

[B7] Garg AX Adhikari NK McDonald H Rosas-Arellan MP Devereaux PJ Beyene J Sam J Haynes RB Effects of computerized clinical decision support systems on practitioner performance and patient outcomes: a systematic review JAMA 2005 293 1223 1238 10.1001/jama.293.10.1223 15755945

[B8] Lobach DF Hammond WE Computerized decision support based on a clinical practice guideline improves compliance with care standards Am J Med 1997 102 89 98 10.1016/S0002-9343(96)00382-8 9209205

[B9] Sucher JF Moore FA Todd SR Sailors RM McKinley BA Computerized clinical decision support: a technology to implement and validate evidence based guidelines J Trauma 2008 64 520 537 10.1097/TA.0b013e3181601812 18301226

[B10] Hirsch MS Brun-Vezinet F Clotet B Conway B Kuritzkes DR D'Aquila RT Demeter LM Hammer SM Johnson VA Loveday C Mellors JW Jacobsen DM Richman DD Antiretroviral drug resistance testing in adults infected with human immunodeficiency virus type 1: 2003 recommendations of an International AIDS Society-USA Panel Clin Infect Dis 2003 37 113 128 10.1086/375597 12830416

[B11] Uy J Brooks JT Baker R Hoffman M Moorman A Novak R HOPS Investigators HIV genotypic resistance testing to optimize antiretroviral prescribing: is there room for improvement? Antivir Ther 2007 12 957 962 17926650

[B12] National Cancer Institute. Breast Cancer Risk Assessment Tool: an interactive tool for measuring the risk of invasive breast cancer http://www.cancer.gov/bcrisktool/default.aspx

[B13] Chaudhry B Wang J Wu S Maglione M Mojica W Roth E Morton SC Shekelle PG Systematic review: impact of health information technology on quality, efficiency, and costs of medical care Ann Intern Med 2006 144 742 752 16702590 10.7326/0003-4819-144-10-200605160-00125

[B14] Dorr D Bonner LM Cohen AN Shoai RS Perrin R Chaney E Young AS Informatics systems to promote improved care for chronic illness: a literature review J Am Med Inform Assoc 2007 14 153 163 10.1197/jamia.M2255 PMC2213468 17213491

[B15] National Cholesterol Education Program, National Heart Lung and Blood Institute. Risk assessment tool for estimating 10-year risk of developing hard CHD (myocardial infarction and coronary death) http://hp2010.nhlbihin.net/atpiii/calculator.asp?usertype=prof

[B16] Deverka PA Doksum T Carlson RJ Integrating molecular medicine into the US health-care system: opportunities, barriers, and policy challenges Clin Pharmacol Ther 2007 82 427 434 10.1038/sj.clpt.6100319 17687271

[B17] McDonald CJ The barriers to electronic medical record systems and how to overcome them J Am Med Inform Assoc 1997 4 213 221 9147340 10.1136/jamia.1997.0040213 PMC61236

[B18] United States Department of Health and Human Services. Health Information Technology: Office of the National Coordinator: Mission http://healthit.hhs.gov/portal/server.pt?open=512&objID=1200&parentname=CommunityPage&parentid=2&mode=2&in_hi_userid=10741&cached=true

[B19] United States Department of Health and Human Services. Personalized health care: opportunities, pathways, resources http://www.hhs.gov/myhealthcare/news/presonalized-healthcare-9-2007.html

[B20] United States Department of Health and Human Services. Personalized health care http://www.hhs.gov/myhealthcare/10.3109/15360288.2015.1037530 26095483

[B21] United States Preventive Services Task Force http://www.ahrq.gov/CLINIC/uspstfix.htm

[B22] Gage B Eby C Johnson J Deych E Rieder M Ridker P Milligan PE Grice G Lenzini P Rettie AE Aquilante CL Grosso L Marsh S Langaee T Farnett LE Voora D Veenstra DL Glynn RJ Barrett A McLeod HL Use of pharmacogenetic and clinical factors to predict the therapeutic dose of warfarin Clin Pharmacol Ther 2008 84 326 331 10.1038/clpt.2008.10 18305455 PMC2683977

[B23] United States Department of Health and Human Services. Health Information Technology: American Health Information Community http://healthit.hhs.gov/portal/server.pt?open=512&objID=1199&parentname=CommunityPage&parentid=7&mode=2&in_hi_userid=10741&cached=true

[B24] United States Department of Health & Human Services. Recommendations for clinical decision support http://healthit.hhs.gov/portal/server.pt/gateway/PTARGS_0_10741_848353_0_0_18/Personalized%20Healthcare%20Workgroup%20Recommendations%20Letter.pdf

[B25] United States Agency for Healthcare Research and Quality. Health information technology: clinical decision support http://healthit.ahrq.gov/portal/server.pt?open=514&objID=5554&mode=2&holderDisplayURL=http://prodportallb.ahrq.gov:7087/publishedcontent/publish/communities/k_o/knowledge_library/key_topics/health_briefing_01242006122700/clinical_decision_support.html

[B26] United States Agency for Healthcare Research and Quality. Health information technology: clinical decision support initiative http://healthit.ahrq.gov/portal/server.pt?open=512&objID=654&&PageID=13665&mode=2&in_hi_userid=3882&cached=true

[B27] Stewart WF Shah NR Selna MJ Paulus RA Walker JM Bridging the inferential gap: the electronic health record and clinical evidence Health Aff (Millwood) 2007 26 w192 w194 10.1377/hlthaff.26.2.w181 17259202 PMC2670472

[B28] Medicare Improvements for Patients and Providers Act of 2008 Pub. L. No. 110-275, 122 Stat. 2494

[B29] Teich JM Osheroff JA Pifer EA Sittig DF Jenders RA Clinical decision support in electronic prescribing: recommendations and an action plan: report of the joint clinical decision support workgroup J Am Med Inform Assoc 2005 12 365 376 10.1197/jamia.M1822 15802474 PMC1174880

[B30] American Recovery and Reinvestment Act of 2009 Pub. L. No. 111-5, 123 Stat. 115

[B31] Health Information Technology Standards Panel http://www.hitsp.org

[B32] Certification Commission for Healthcare Information Technology http://www.cchit.org

